# Interactive Effects of Two Global Contaminants on Behavioral Variation in Brine Shrimp

**DOI:** 10.1002/ece3.73495

**Published:** 2026-04-16

**Authors:** Marta Favero, Bianca Melita Palmas, Giulia Forte, Karina C. Lau, Anisa Bardhi, Andrea Barbarossa, Marialetizia Palomba, Daniele Canestrelli, Giovanni Polverino

**Affiliations:** ^1^ Department of Ecological and Biological Sciences University of Tuscia Viterbo Italy; ^2^ Department of Veterinary Medical Sciences University of Bologna Bologna Italy

**Keywords:** activity, antagonistic interaction, behavioral ecotoxicology, behavioral individuality, behavioral plasticity, pollutant mixtures

## Abstract

Polycyclic aromatic hydrocarbons and chlorophenols are common aquatic pollutants from industrial and agricultural runoff that have adverse effects on living organisms and aquatic ecosystems in general. While aquatic wildlife is often exposed to these contaminants simultaneously, little is known about their combined effects. To fill this gap, we tested whether and how exposure to relevant concentrations of phenanthrene (Phe), 2,4‐dichlorophenol (2,4‐DCP), or a mixture of both altered key behaviors in brine shrimp ( *Artemia parthenogenetica* ), an emerging model in behavioral ecotoxicology. We exposed newborns to one of four treatments until adulthood: control, Phe (400 ng/L), 2,4‐DCP (400 ng/L), and the combination of both Phe and 2,4‐DCP (400 ng/L each). We repeatedly measured individual activity levels at adulthood in both the x‐y plane (distance moved) and in three dimensions (mobility), as suggested for planktonic animals. We found that Phe, but not 2,4‐DCP, significantly reduced average activity levels that were better captured by looking at activity in three than two dimensions. When combined, the pollutants appeared to exhibit an antagonistic interaction, with 2,4‐DCP mitigating phenanthrene's negative impact on average mobility levels. Moving beyond average effects, we tested whether effects also appeared at the individual level. Our analysis revealed that within‐individual variation (behavioral plasticity) in mobility decreased substantially in all exposure treatments compared to unexposed animals, whereas between‐individual variation (behavioral individuality) did not vary. Our findings highlight the importance of considering interactive and individual‐level effects of pollutants in ecological risk assessment, as effects may differ from predictions based on single contaminants and average responses alone.

## Graphical abstract

Wildlife is often exposed to multiple pollutants with unknown joint effects. We tested how two aquatic pollutants at realistic levels alter activity in Artemia. Phenanthrene reduced average activity but not when mixed with 2,4‐dichlorophenol. Both pollutants and their mix reduced behavioral plasticity but not individuality, highlighting the importance of considering interactive and individual‐level effects of pollutants in ecological risk assessments.

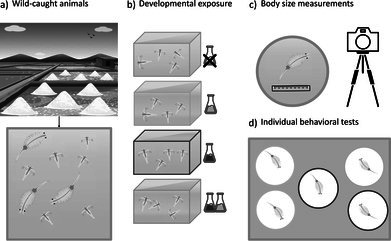

## 1 Introduction

Aquatic ecosystems are increasingly exposed to a wide range of human‐released chemicals, including organic compounds, pharmaceuticals, and pesticides produced by industrial discharges, urban wastewater, and agricultural runoff. These compounds pose a significant threat to aquatic organisms globally, having negative impacts on their behavior, growth, reproduction, and survival (Pyle and Ford 2017; Bertram et al. 2022). However, classic studies in behavioral ecotoxicology typically focus on single contaminants, often neglecting their natural cocktails (Saaristo et al. 2018; Brack et al. 2019; Bertram et al. 2022). This is important since real‐world scenarios are often complex (Pyle and Ford 2017), and multi‐stressor exposures reflect most environmental conditions in polluted habitats (Breitburg et al. 1998; Hale et al. 2017; Orr et al. 2020; Bertram et al. 2022). In fact, although interactive effects of environmental contaminants have traditionally been assumed to be additive, increasing evidence suggests that stressors can also interact synergistically or antagonistically, producing unexpected ecological outcomes (Folt et al. 1999; Ban et al. 2014; Piggott et al. 2015; Hale et al. 2017; Aulsebrook 2023). For instance, a recent study found that the mixture of pharmaceuticals commonly found in the environment—painkillers, diabetes medications, and estrogen hormones—and the antibiotic ciprofloxacin greatly increased the resistance of bacterial communities to multiple antibiotics, even some that are chemically different from ciprofloxacin (Hayes et al. 2025). Evidence that pollutant mixtures in waterways can trigger unexpected responses in exposed wildlife underscores the limits of predicting impacts from single contaminants alone.

A primary strategy by which animals cope with environmental pollution is through behavioral adjustments (Sih 2013; Wong and Candolin 2015). A growing body of literature indicates that ecologically relevant behaviors are often altered in animals exposed to single pollutants, but evidence on the effects of exposure to multiple contaminants simultaneously is scarce and shows mixed results (Hale et al. 2017). For example, in amphibian larvae ( *Limnodynastes tasmaniensis* ), exposure to copper or the insecticide imidacloprid reduced swimming activity and escape responses, respectively, with an interactive effect of the two stressors observed only for erratic swimming behavior (Sievers et al. 2018). Mosquitofish ( *Gambusia holbrooki* ) exposed to the hormonal contaminant 17β‐trenbolone increased their exploration and decreased their predator‐escape behavior, with these effects being temperature‐dependent (Lagesson et al. 2019). Moreover, mosquito larvae ( *Culex pipiens* ) lowered their antipredator responses when exposed to either the pesticide chlorpyrifos or heat stress, but not when exposed to bot stressors together (Meng et al. 2021). A similar result was also found for the reproductive and activity behavior of guppies ( *Poecilia reticulata* ), which varied only in animals exposed to either heat stress or the anxiolytic contaminant fluoxetine (Wiles et al. 2020). The limited number of empirical studies and their contrasting evidence highlight the need for further analysis on the effects of multiple stressors on wildlife behavior, including effects of a mixture of environmental contaminants (Halfwerk and Slabbekoorn 2015; Hale et al. 2017; Jacquin et al. 2020). Behavioral changes resulting from exposure to environmental contaminants can negatively impact wildlife and entire ecosystems, as recently observed for the migratory behaviors of wild Atlantic salmon ( *Salmo salar* ) in response to pharmaceutical pollution (Brand et al. 2025).

On one side, we know that chemical contaminants can affect animal behavior at the population level (reviewed in Saaristo et al. 2018). On the other side, whether all individuals of a population are equally vulnerable to such contaminants remains largely unknown (Saaristo et al. 2018; Bertram et al. 2022). This is important because variation in behavior between individuals (i.e., behavioral individuality; Réale et al. 2007) and within individuals (i.e., behavioral plasticity; Dingemanse and Wolf 2013) are essential for populations to cope with environmental challenges and adapt to a rapidly changing world (Wong and Candolin 2015). Recent evidence indeed suggests that the deleterious effects of environmental pollutants on wildlife behavior may extend beyond mean responses (Polverino et al. 2021). For example, exposure to anxiolytic contaminants has been shown to reduce between‐individual variation in guppies ( *Poecilia reticulata* ; Tan et al. 2020; Polverino et al. 2021, 2023), within‐individual variation in snails ( *Physa acuta* ) and female guppies (Henry et al. 2022; Polverino et al. 2023), and even alter correlations between behavioral and life‐history traits in guppies (Aich et al. 2025). However, only a limited number of studies have tested the impact of global contaminants on individual‐level variation in animal behavior, and most of those have focused on pharmaceutical contaminants (but see Favero et al. 2025). Yet none have tested the individual‐level effects of exposure to multiple contaminants simultaneously. Focusing on these aspects will therefore increase environmental realism, revealing how natural cocktails of pollutants affect populations in the wild (Bertram et al. 2022).

To fill this gap, we tested whether and how environmentally relevant concentrations of two global contaminants—phenanthrene (Phe; a polycyclic aromatic hydrocarbon, PAH), 2,4‐dichlorophenol (2,4‐DCP; a chlorinated phenol derivative), and their mixture—altered mean behavioral traits, between‐individual variation, and within‐individual variation in brine shrimp, an emerging model in behavioral ecotoxicology research (Persoone and Wells 1987; Libralato et al. 2016; Favero et al. 2025). These crustaceans inhabit hypersaline environments worldwide, including salt marshes, wetlands, and salty lakes, which are increasingly contaminated by human‐derived pollutants, including PAHs and chlorophenols (Triantaphyllidis et al. 1998; Kot‐Wasik et al. 2004; Cong et al. 2021). Among these contaminants, phenanthrene and 2,4‐dichlorophenol are particularly concerning because they are globally spread and are toxic to living organisms (Phe: Mojiri et al. 2019; Honda and Suzuki 2020; 2,4‐DCP: Jin et al. 2011; Ma et al. 2012; Wang et al. 2020). They originate respectively from the incomplete combustion of organic matter such as wood and fuels (Boström et al. 2002) and from the production of herbicides (Dimou et al. 2006). Their concentrations in the wild have been reported to range from ng/L in surface waters to μg/L in coastal waters (Peng et al. 2019 and references therein; Dimou et al. 2006; Gao et al. 2008). Phenanthrene's toxicity is partly due to the inhibition of acetylcholinesterase, a key enzyme for muscle contraction (Gauthier et al. 2016; Aguilar et al. 2020; Turja et al. 2020). Instead, 2,4‐dichlorophenol is an endocrine disruptor of energy metabolism (Shannon et al. 1991; Penttinen 1995; Penttinen et al. 1996). They can both affect animal behavior even at low concentrations (Phe: Correia et al. 2007; Torreiro‐Melo et al. 2015; 2,4‐DCP: Kaiser et al. 1995; Tessier et al. 2000; Bahrndorff et al. 2016; Ferris Pasquini et al. 2023), and phenanthrene was even found to alter within‐ and between‐individual variation in brine shrimp behavior (Favero et al. 2025). Despite the frequent co‐occurrence of phenanthrene and 2,4‐dichlorophenol in aquatic environments, their combined effects on exposed wildlife remain unknown.

Our study fills this gap by examining whether and how environmentally realistic concentrations of these pollutants, alone and in combination, alter mean behavioral traits, as well as within‐ and between‐individual variation in *Artemia parthenogenetica* . We predict that exposure to phenanthrene and 2,4‐dichlorophenol will reduce mean activity levels and impair individual‐level behavior in the exposed groups, with stronger effects observed in response to simultaneous exposure to both contaminants.

## 2 Methods

### 2.1 Ethical Note

In this study, we used crustaceans, which do not require animal welfare permits. However, we observed the general principles outlined in the ASAB/ABS guidelines for the ethical treatment of animals in research, ensuring that our experimental procedures were designed to minimize stress.

### 2.2 Experimental Animals

**FIGURE 1:**
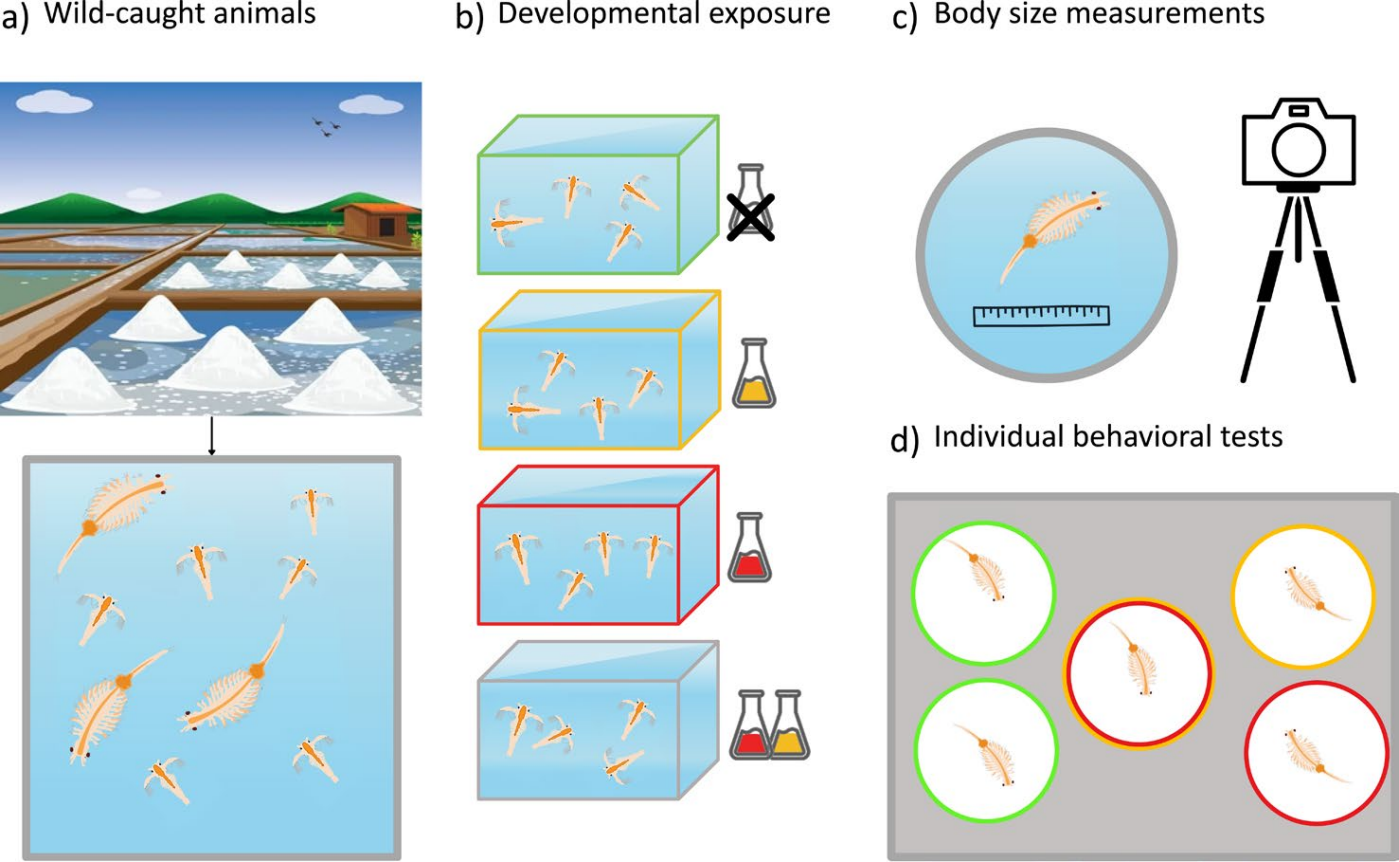
Schematic of the experimental design. (a) Newly hatched larvae (nauplii) of *Artemia parthenogenetica* descending from wild animals collected at Salina dei Monaci (Southern Italy) were distributed across twelve independent tanks and randomly assigned to four exposure treatments. (b) The exposure period lasted 30 days, from the nauplius stage to adulthood. Tank color indicates exposure treatment: green (Ctrl), yellow (Phe at 400 ng/L), red (2,4‐DCP at 400 ng/L), and gray (Mix of 400 ng/L of each contaminant). (c) At adulthood, individuals were first photographed for body size measurements. (d) Afterwards, behavioral assays were conducted in an open‐field setup, with five arenas tested in parallel. Activity levels were recorded daily over three consecutive days and scored, blind to the treatment, with the tracking software EthoVision XT. A total of 232 individuals completed all behavioral trials (Ctrl: *N* = 58, Phe: *N* = 56, 2,4‐DCP: *N* = 59, Mix: *N* = 59).

The experimental animals used in this study represented the twelfth overlapping generation descended from the wild‐caught *A. parthenogenetica* (Browne and Wanigasekera 2000) collected from the natural reserve Salina dei Monaci (Southern Italy; 40°18′8″ N, 17°43′55″ E) and subsequently transferred into large mesocosms in our laboratory (Figure 1a). The large mesocosms (100 L capacity) were filled with water and sediment from the natural sampling site and maintained at controlled temperatures (28°C) and salinity (between 80 and 120 g/L), equipped with air probes to guarantee proper water oxygenation, and exposed to natural light and photoperiod conditions.

Newly hatched larvae (nauplii) and water were randomly collected from the large mesocosms and distributed across twelve independent experimental tanks (2 L). These tanks were housed in a temperature‐controlled chamber set at 26°C ± 0.5°C with a 12:12 h light/dark photoperiod for the duration of the study. The animals were fed twice a week with 4 mL of green microalgae (*Nannochloropsis* sp.).

### 2.3 Treatments

The twelve experimental tanks, each containing approximately 20 nauplii, were randomly assigned to four treatment groups: (1) a control group not exposed to contaminants (Ctrl), (2) a group exposed to 400 ng/L of phenanthrene (Phe), (3) a group exposed to 400 ng/L of 2,4‐dichlorophenol (2,4‐DCP), and (4) a group exposed to a mixture of 400 ng/L of phenanthrene and 400 ng/L of 2,4‐dichlorophenol (Mix). Exposure began shortly after hatching and continued throughout the developmental period until adulthood (30 days; Figure 1b).

The two pollutants were selected based on preliminary analyses conducted in a natural brine shrimp habitat in Central Italy, with the aim of assessing the potential contamination levels to which these ecosystems are exposed (see Table S1 in Supporting Information). Nonlethal concentrations of these two contaminants were selected between the maximum concentrations at which no negative effects are observed (NOEC) and the minimum concentration at which effects appeared in the exposed organisms (LOEC; EPA 1994). These environmentally relevant concentrations also match those reported in waterways globally (Phe: Peng et al. 2019 and references therein; 2,4‐DCP: Dimou et al. 2006; Gao et al. 2008).

We also analyzed water samples from our own experimental tanks after the behavioral trials to verify the actual concentrations experienced by the animals. In general, concentrations of both pollutants were reduced to approximately one quarter of their initial levels (see Table S2 in Supporting Information). Water samples were stored at −20°C until chemical analysis was performed. Phenanthrene concentration was determined with gas chromatography coupled with mass spectrometry (GC–MS), whereas 2,4‐dichlorophenol was measured using liquid chromatography coupled with tandem mass spectrometry (LC–MS/MS). A detailed description of the analytical procedure is provided in the Supporting Information.

### 2.4 Procedure

Upon reaching sexual maturity, 20 individuals from one of the three experimental tanks in each treatment were randomly selected and photographed for body size measurements. Individuals were then transferred into individual 0.100 L flasks filled with fresh treatment water for the subsequent individual behavioral tests. Individual flasks were housed in a separate temperature‐controlled chamber at 26°C ± 0.5°C with a 12:12 h light/dark cycle for the whole duration of the behavioral tests. Behavioral assays were conducted in three consecutive experimental rounds, with 80 individuals (20 per treatment) in each round and a total of 240 individuals. Each individual performed three behavioral tests (24 h apart on three consecutive days; Figure 1).

### 2.5 Body Size and Behavioral Measurements

#### 2.5.1 Body Size

Prior to behavioral tests, each individual was placed in a Petri dish next to graph paper and photographed with a digital camera (OLYMPUS Tough TG‐5; 12MP BSI CMOS sensor, 25–100 mm F2‐4.9 lens) on a tripod placed at 15 cm distance (see Figure 1c). The total length, from head to the end of the abdomen (Thoré et al. 2021; Favero et al. 2025), was then measured using the software ImageJ (Schneider et al. 2012) from each photo, which had a fixed, known scale (1 mm = 3.779 pixels). Body size was measured and included as a covariate in the behavioral models to account for behavioral variation explained by differences in the size of the animals.

#### 2.5.2 Open Field Test

After a 24‐h acclimatization period in the isolation flasks, three separate behavioral tests were conducted on each animal, once a day, between 10 a.m. and 4 p.m., to capture variability in activity performance associated with the diurnal behavioral patterns. Animals were fed *ad libitum* with *Nannochloropsis* sp. algae at the end of each testing day.

To quantify adult behaviors, we randomly selected five individuals at a time from the pool of 20 isolated individuals within each treatment group and placed them individually in the experimental arenas for behavioral testing (Figure 1d). This procedure was repeated until all 20 individuals per treatment were tested. Each arena consisted of an open circular field with a 5.5 cm diameter and a white background containing 9 mL of treatment water. After a 1‐min habituation, we recorded the activity for 10 min using a GigE monochrome camera (1/1.8′ CMOS sensor; 1280 × 1024, max. 60 fps) with a Kowa lens (4.4–11 mm), mounted on a tripod 40 cm above the arena and connected to the EthoVision XT video‐tracking software (version 17.5.1718, Noldus Information Technology).

Behavioral variation in activity levels is crucial in non‐sexile species and can have ecological and evolutionary implications (Réale et al. 2007; Wolf and Weissing 2012). Therefore, we quantified individual activity levels with an open field test (Réale et al. 2007; Montiglio et al. 2010) by measuring: distance moved (in mm) and movement (i.e., time spent moving in s) with a starting velocity threshold set at 5.000 mm/s. This threshold corresponds to the minimum body length measured in our experimental animals. In other words, an individual was classified as moving when its velocity exceeded 5.000 mm/s and as not moving when its velocity was below 4.500 mm/s. Moreover, since planktonic organisms often live suspended in the water column and their movement is not limited to the x‐y plane, we measured the mobility state of the animals to also account for three‐dimensional movements such as rotations, vibrations, and rolling or folding of their body (Dodson et al. 1997; Kwak and Bae 2018). Mobility state was classified into two levels: low mobility and mobility. This classification was determined based on the percentage of modified pixels, representing changes in the animal's outline detected between consecutive samples taken 3 msec apart (mean interval = 1 sample), as well as their duration in s (Grieco et al. 2010). These thresholds were determined empirically using the Integrated Visualization function in EthoVision XT to observe individual activity (Figure S1 in the Supporting Information). Individuals were classified as mobile when the percentage of modified pixels was above 25%, while low mobility referred to values below 25%, as per (Favero et al. 2026).

### 2.6 Statistical Analysis

A total of 232 out of 240 individuals completed all behavioral trials (control: *N* = 58; phenanthrene: *N* = 56; 2,4‐dichlorophenol: *N* = 59; and the mixture of both contaminants: *N* = 59), resulting in 707 behavioral tests (three per individual) and ~27 h of video recordings. Eight individuals (Ctrl: *N* = 2; Phe: *N* = 4; 2,4‐DCP: *N* = 1; Mix: *N* = 1) did not complete all the behavioral tests due to early mortality. Data were analyzed using *R* version 4.2.2 (R Core Team 2022) with the statistical packages *lmerTest*, *MCMCglmm*, and *emmeans* (Hadfield 2010; Kuznetsova et al. 2017; Lenth et al. 2024).

We tested whether exposure to each pollutant and their mix affected mean behaviors of brine shrimp by fitting linear mixed‐effect models (LMMs; Bolker et al. 2009) with distance moved, movement (i.e., time spent moving), mobility, and low mobility one‐by‐one as the dependent variables. Fixed effects included treatment (Ctrl, Phe, 2,4‐DCP, and Mix), trial (three repeated measures per individual), recording time (up to 600 s), and experimental arenas (*N* = 5). Individual identity and experimental round were included as random intercepts to account for repeated measures and temporal grouping, while trial and round were included as random slopes.

We also tested whether mean behaviors differed across treatments depending on the body size of the animals. To do so, we included the interaction between body size and treatment in each model and used a model reduction approach to test whether our model fit better when this interaction was included. Our analysis revealed that the interaction between body size and treatment never explained a significant portion of the behavioral variance observed, and therefore we did not include it in the final models.

The response variables were squareroot transformed to meet the assumptions of normality. First, to determine whether random intercepts explained a significant proportion of the observed variation, we compared a full model including individual identities as a random effect with a reduced model without this term. The model comparison was assessed using likelihood‐ratio tests (LRTs) and Akaike Information Criterion differences (ΔAIC). Models with lower AIC were preferred, with ΔAIC ≥ 2 and significant LRTs (*p* < 0.05) interpreted as meaningful support for the more complex model. We also tested whether individuals varied their behavior across rounds and trials. To do so, we ran different models for each behavioral trait with individuals and round as random intercepts and round and/or trial as random slopes. We then compared the models with random slopes and intercepts to those with only random intercepts using LRTs and ΔAICs. We assumed error distribution to be normal and checked it by visually inspecting the model residuals, although LMMs are robust to violations of distributional assumptions (Schielzeth et al. 2020). Statistical significance was set at α < 0.05.

To test whether and how within‐ and between‐individual variation in behavior varied across treatments, we partitioned behavioral variation into within‐individual (residuals) and between‐individual (intercepts) components using the Markov Chain Monte Carlo method. Posterior distributions were generated using weakly informative priors (Lemoine 2019). For the residual variance, we used a weakly informative inverse‐Wishart prior (V = 10, ν = 3), while for the random effect variances (G1, G2) we chose scaled inverse‐Wishart priors (V = 50, ν = 5 for G1; V = 1, ν = 1 for G2), which provide greater flexibility while preserving the relationship between variances and correlations (Gelman and Hill 2007; Alvarez et al. 2014). Simulations were conducted with 1,500,000 resamplings, 500,000 burn‐ins, and thinning every 100 iterations. Posterior density plots were visually inspected to ensure model convergence. Variance components and repeatability estimates derived from the models were used for pairwise comparisons. Significant differences were identified when 95% credible intervals of the pairwise comparisons did not overlap with zero.

## 3 Results

### 3.1 Mean Effects

**FIGURE 2:**
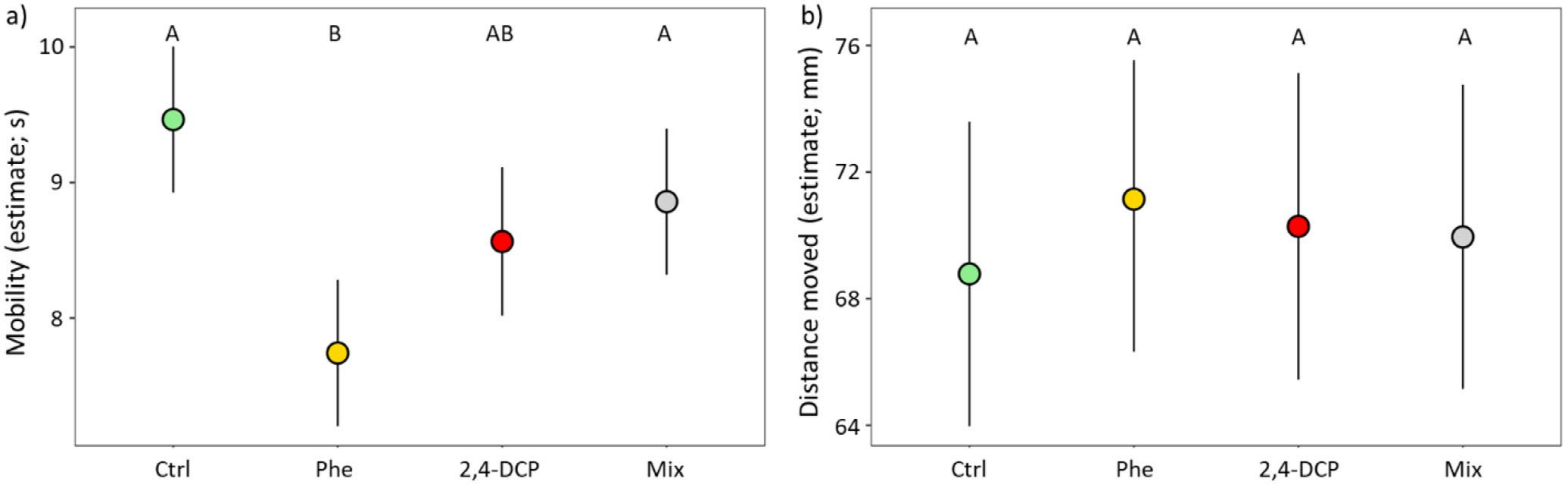
Estimated marginal means (± SE) for mobility (a) and distance moved (b) across exposure treatments (Ctrl: *N* = 58; Phe: *N* = 56; 2,4‐DCP: *N* = 59; Mix: *N* = 59). Points represent model‐adjusted means controlling for covariates included in the models. Pairwise comparisons between treatment groups were adjusted with the Bonferroni method. Treatments not sharing the same letter are significantly different (*p* < 0.05).

We found a significant effect of treatment on mobility levels (*p* < 0.001), with a reduction in mobility in animals exposed to phenanthrene compared to controls (Ctrl‐Phe: estimate ± SE = 1.724 ± 0.405; df_218_; *p* < 0.001; Figure 2a) and the group exposed to both pollutants simultaneously (Mix‐Phe: estimate ± SE = 1.118 ± 0.404; df_229_; *p* = 0.036; Figure 2a). On the contrary, animals exposed to 2,4‐DCP did not differ in their mobility compared to other treatments (*p* > 0.05; Figure 2a).

Larger animals were on average more mobile than smaller ones (*p* = 0.025; Table 1) and in general mobility increased across trials (*p* < 0.001; Table 1). In contrast, we did not find significant differences in distance moved across treatments (*p* > 0.05; Figure 2b). However, body size had a positive effect on the distance moved (*p* = 0.027; Table 1). To avoid redundancy, results for low mobility and movement (i.e., time spent moving) are reported in the Supporting Information (Table S3; Figures S2 and S3), since these measures are complementary to mobility and, in fact, showed the same treatment patterns.

**TABLE 1:** Results from the models for mobility and distance moved as the dependent variables.

**Mobility (s)**						
*Fixed effects*	*Sum sq*	*Mean sq*	*NumDF*	*DenDF*	*F value*	*p*
Treatment	135.860	45.290	3	217.280	6.666	**< 0.001**
Body size	34.420	34.420	1	217.780	5.067	**0.025**
Trial	554.190	554.190	1	401.980	81.577	**< 0.001**
Arena	398.180	99.540	4	590.410	14.653	**< 0.001**
Time in zone	29.720	29.720	1	490.160	4.374	**0.037**
*Random effects*	*Estimate*	*AIC*	*BIC*	*LogLik*	*Chisq*	*p*
V_between_	10.868					
V_within_	6.793					
Repeatability	0.615	3171.900	3234.100	−1571.900	46.322	**< 0.001**
Round.slope	1.850	3169.900	3241.000	−1569.000	5.945	**0.051**
Trial.slope		3173.500	3244.600	−1570.800	2.316	0.314
Both slopes		3169.900	3249.900	−1567.000	9.949	**0.041**
**Distance moved (mm)**						
*Fixed effects*	*Sum Sq*	*Mean Sq*	*NumDF*	*DenDF*	*F value*	*p*
Treatment	248.500	82.840	3	233.650	0.297	0.828
Body size	1386.300	1386.320	1	240.530	4.962	**0.027**
Trial	1599.400	1599.420	1	472.710	5.724	**0.017**
Arena	3314.500	828.610	4	684.660	2.966	**0.019**
Time in zone	524.000	524.050	1	674.450	1.876	0.171
*Random effects*	*Estimate*	*AIC*	*BIC*	*logLik*	*Chisq*	*p*
V_between_	24.900					
V_within_	279.420					
Repeatability	0.082	6044.900	6108.700	−3008.400	40.480	**< 0.001**
Round.slope		6048.300	6121.300	−3008.200	0.531	0.767
Trial.slope		6044.600	6117.600	−3006.300	4.231	0.121
Both slopes		6044.100	6126.100	−3004.000	8.802	0.066

*Note:* Fixed effects in the models included treatment (Ctrl, Phe, 2,4‐DCP, Mix), body size, trial (three repeated measures per individual), experimental arenas (*N* = 5), and recording time (up to 600 s). Random effects included random intercepts (individuals and rounds) and slopes (rounds and trials). All models included individual identities as random intercepts, which allowed variance partitioning: between‐individual variation (intercepts; V_between_), within‐individual variation (residuals; V_within_), and their proportion (behavioral repeatability). Test statistics and significance levels for random effects were determined by comparing full models to null models (excluding intercepts or slopes) using likelihood ratio tests and Akaike Information Criterion. The significance level was set at α < 0.05, with significant results represented in bold.

### 3.2 Individual‐Level Effects

**FIGURE 3:**
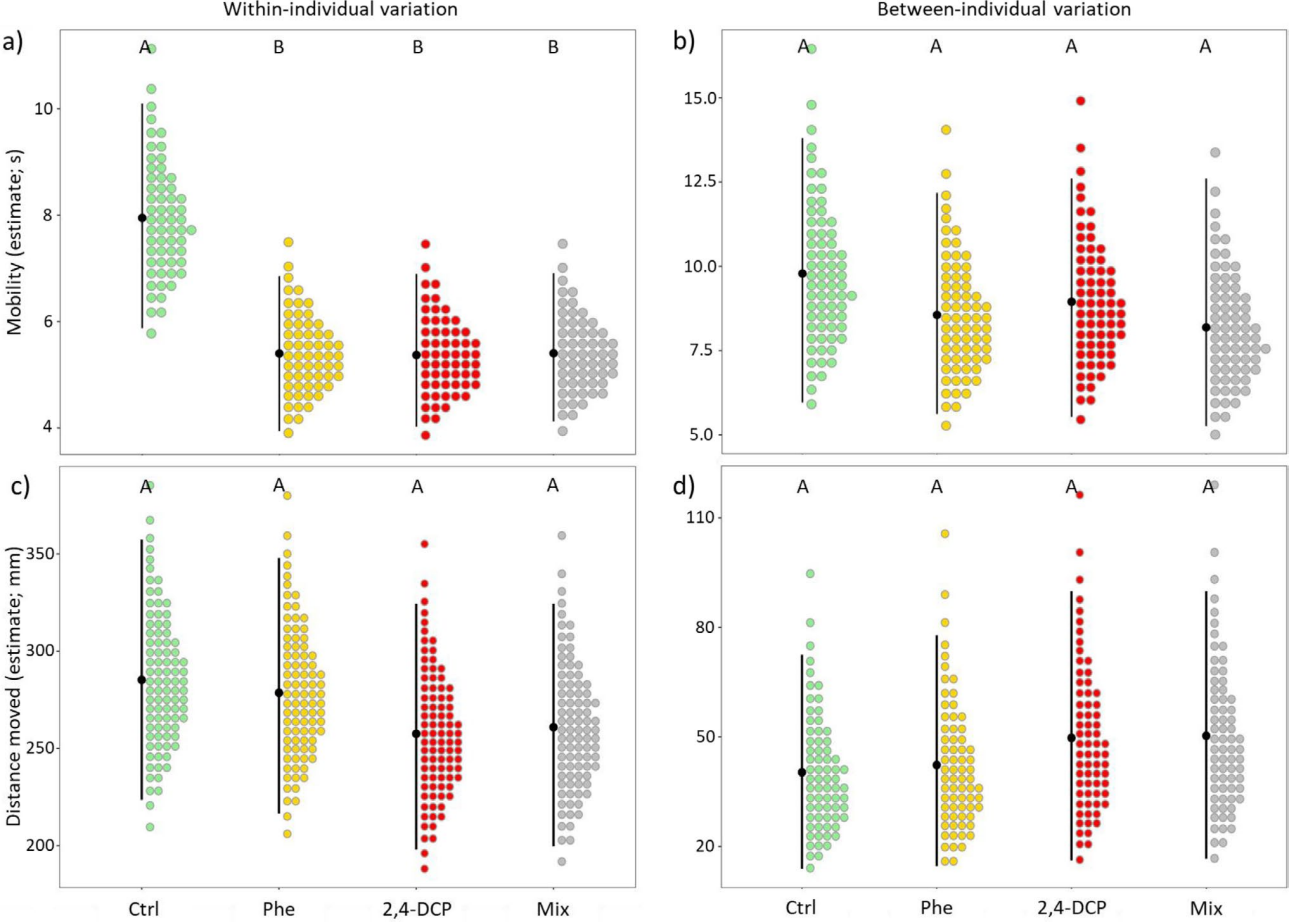
Behavioral variation within individuals (behavioral plasticity; a, c) and between individuals (behavioral individuality; b, d) in mobility and distance moved across exposure treatments (Ctrl: *N* = 58; Phe: *N* = 56; 2,4‐DCP: *N* = 59; Mix: *N* = 59). For each plot, black points represent mean variance estimates, vertical lines represent the 95% credible intervals, and colored dots represent probability densities.

We found that within‐individual variation in mobility levels was substantially lower in animals exposed to either phenanthrene, 2,4‐dichlorophenol, or their mix than in unexposed animals (Figure 3a). However, no differences in within‐individual variation were observed for distance moved across treatments (Figure 3c). In contrast, we found no significant differences in between‐individual variation in both mobility (Figure 3b) and distance moved (Figure 3d) across the exposure treatments.

## 4 Discussion

This study explored the effects of environmentally realistic concentrations of two global contaminants—phenanthrene, 2,4‐dichlorophenol, and their mixture—on both mean behaviors and individual variation in the behavior of *Artemia parthenogenetica* . By quantifying mean mobility and distance moved and partitioning their variance into the within‐ and between‐individual components, our results revealed that sublethal exposures to phenanthrene reduced mean mobility levels, while within‐individual variation in mobility was lower in all exposure treatments compared to controls. In contrast, between‐individual variation did not differ among treatments for either mobility or distance moved.

A key finding of this study is the reduction in mean mobility levels (time spent moving) observed in animals exposed to phenanthrene but not to both phenanthrene and 2,4‐dichlorophenol simultaneously. Lower activity levels in response to phenanthrene exposure are in line with evidence from previous studies on aquatic species, from invertebrates (Costa et al. 2016; Gauthier et al. 2016) to fishes (Correia et al. 2007; Torreiro‐Melo et al. 2015). This alteration of activity‐related behaviors is concerning since it can influence both the ecological and evolutionary processes of wild populations, as recently observed by Brand et al. (2025) with respect to the migratory behaviors of wild Atlantic salmon ( *Salmo salar* ) exposed to psychoactive pollution. Yet our analysis showed that small, ecologically relevant concentrations of 2,4‐dichlorophenol did not alter the mean activity levels (mobility and distance moved) of our brine shrimp. This contrasts with evidence from studies on *Daphnia magna* exposed, however, to much higher concentrations of this pollutant, ranging from 0.4 to 3.6 mg/L (Bahrndorff et al. 2016). Therefore, it is possible that the lack of behavioral effects observed in our study resulted from the lower concentration of 2,4‐dichlorophenol, though environmentally relevant and therefore likely to reflect real‐world scenarios. Alternatively, it is possible that brine shrimp are more resistant to environmental perturbations, including contaminants, than freshwater invertebrates such as *Daphnia magna* (Nunes et al. 2006), as brine shrimp are already adapted to thrive in extreme environments. Another, non‐mutually exclusive explanation is that 2,4‐dichlorophenol might be less toxic than phenanthrene, with the latter, in fact, known to have negative effects already at very low concentrations (Peng et al. 2019; Cong et al. 2021).

Interestingly, the simultaneous exposure to both pollutants did not result in noticeable changes in the mean activity levels (mobility and distance moved) of our shrimp compared to control conditions. This suggests that these pollutants may have had an antagonistic interaction, although more tests are needed to verify whether this is the case. In other words, 2,4‐dichlorophenol reduced the negative effects of phenanthrene on mean mobility, contrary to the additive or synergistic effects that are typically expected from pollutant interactions (Piggott et al. 2015; Hale et al. 2017). A possible explanation is that the impact of phenanthrene on brine shrimp behavior was masked by physiological compensation triggered by the concurrent exposure to 2,4‐dichlorophenol. In fact, phenanthrene is known to reduce the activity of the enzyme acetylcholinesterase and compromise muscle contraction: at high dosages, phenanthrene can even lead to immobility and respiratory failure (Zhang et al. 2014; Chen et al. 2025). In contrast, exposure to 2,4‐dichlorophenol was found to compromise energy production at the cell level, resulting in compensatory changes in metabolism through increased respiration rates (Shannon et al. 1991; Penttinen 1995). Since higher metabolic rates are often positively correlated with higher activity levels in living organisms (Sih et al. 2015), it is possible that 2,4‐dichlorophenol might have buffered the negative effects of phenanthrene on mean mobility, which were, in fact, no longer detected when animals were exposed to the cocktail of both contaminants. These findings emphasize the importance of considering contaminant interactions in ecological risk assessments (discussed in Ågerstrand et al. 2020), as combined effects can substantially differ from predictions based on contaminants tested one‐by‐one.

A second major finding of our study was the reduction in within‐individual variation in mobility observed across all exposed groups compared to unexposed animals. Our findings are consistent with previous research showing that environmental pollutants can compromise within‐individual variation in wildlife behavior, as observed in *Artemia franciscana* exposed to phenanthrene (Favero et al. 2025), freshwater snails ( *Physa acuta* ; Henry et al. 2022) and guppies ( *Poecilia reticulata* ; Polverino et al. 2023; Aich et al. 2025) exposed to fluoxetine, and hermit crabs ( *Pagurus bernhardus* ; Nanninga et al. 2020) exposed to microplastics. This reduction in within‐individual variation is concerning, as it constrains an individual's ability to adjust to environmental challenges such as predation, fluctuations in resource abundance, and environmental changes in general (Dingemanse and Wolf 2010; Westneat et al. 2015; Wong and Candolin 2015). Within‐individual variation enables individuals to optimize survival strategies in response to changing conditions (Dingemanse and Wolf 2013; Snell‐Rood 2013), although sustaining the sensory and regulatory systems necessary for high responsiveness imposes considerable energetic costs (DeWitt et al. 1998). Under this perspective, it is reasonable to assume that living in polluted habitats might require individuals to allocate energy across competing functions, prioritizing investments in stress mitigation and homeostasis over behavioral plasticity (Westneat et al. 2015). In fact, chlorophenols can increase heat generation in exposed organisms, therefore increasing the energy needed for thermoregulation (Penttinen and Kukkonen 1998). In addition, it is possible that the reduction in within‐individual variation we observed is linked to a pollution‐induced impairment of cognitive abilities, which has already been described as a collateral effect of phenanthrene on exposed animals (Chen et al. 2019; Olasehinde and Olaniran 2022). The comparable reduction in within‐individual variation we found in mobility but not in distance moved in animals exposed to phenanthrene, 2,4‐dichlorophenol, and their mix highlights the complexity of interacting stressors and their unpredictable outcomes (Hale et al. 2017; Bertram et al. 2022). In sharp contrast, and contrary to our assumption, exposure to phenanthrene, 2,4‐dichlorophenol, and their combination had no significant impact on between‐individual variation in activity levels. This result, however, aligns with studies on other aquatic species such as freshwater snails and guppies, which were found to maintain their between‐individual variation after long‐term exposures to psychoactive pollutants (Henry et al. 2022; Aich et al. 2025). The consistent between‐individual variation observed across our exposure treatments suggests that this species may be well adapted to cope not only with the variable conditions typical of its extreme habitat (Sih 2013)—such as high salinity and pronounced temperature fluctuations—but also with the presence of pollutants.

Overall, our evidence indicates that phenanthrene, but not 2,4‐dichlorophenol, reduces mean mobility in *A. parthenogenetica* and that both contaminants, either alone or in combination, compromise within‐individual variation in mobility. These findings highlight the unpredictable effects that cocktails of environmental pollutants can have on animal behavior. Understanding the collateral effects of global pollutants on both mean behavioral traits and within‐ and between‐individual variation in animal behavior is critical to predict the resilience of populations to their increasingly contaminated ecosystems.

## Author Contributions

**Marta Favero:** conceptualization (equal), formal analysis (supporting), methodology (supporting), validation (equal), visualization (supporting), writing – review and editing (equal). **Bianca Melita Palmas:** data curation (equal), formal analysis (lead), methodology (equal), validation (equal), visualization (lead), writing – original draft (equal). **Giulia Forte:** data curation (equal), formal analysis (supporting). **Karina C. Lau:** data curation (equal), formal analysis (supporting). **Anisa Bardhi:** data curation (equal), formal analysis (supporting). **Andrea Barbarossa:** data curation (equal), formal analysis (supporting). **Marialetizia Palomba:** data curation (supporting), methodology (supporting), resources (equal). **Daniele Canestrelli:** funding acquisition (supporting), resources (equal). **Giovanni Polverino:** conceptualization (lead), formal analysis (equal), funding acquisition (lead), investigation (lead), project administration (lead), resources (equal), supervision (lead), validation (supporting), writing – original draft (equal), writing – review and editing (equal).

## Funding

The work was supported by a Young Independent Research Group award (to M.P. and G.P.) funded by the University of Tuscia. The project was implemented under the National Recovery and Resilience Plan (NRRP), Mission 4 Component 2 Investment 1.4—Call for tender No. 3138 of 16 December 2021, rectified by Decree n.3175 of 18 December 2021 of the Italian Ministry of University and Research funded by the European Union—Next Generation EU. Project code CN_00000033, Concession Decree No. 1034 of 17 June 2022 adopted by the Italian Ministry of University and Research, CUP J83C22000860007, Project title “National Biodiversity Future Centre—NBFC”; and Mission 4 Component 2 Investment 3.1.—Italian Ministry of University and Research funded by the European Union—NextGenerationEU; Project code IR0000035, CUP C63C22000570001, Project title “Unlocking the Potential for health and food from the seas”—EMBRC UP.

## Conflicts of Interest

The authors declare no conflicts of interest.

## Supporting information


**Table S1:** Presence and concentrations of phenanthrene and 2,4‐dichlorophenol in seawater samples collected from a local natural brine shrimp habitat and adjacent coastal seawaters in Central Italy. Geographic coordinates of sites, dates, and time of collection are reported.
**Table S2:** Concentrations of phenanthrene (Phe) and 2,4‐dichlorophenol (2,4‐DCP) in the water collected from the experimental tanks at the end of the behavioral tests.
**Table S3:** Results from the models for low mobility and movement as the dependent variables.
**Figure S1:** Snapshot of the Integrated Visualization tool in EthoVision XT used to select threshold values for movement and mobility, and to assess the difference between these two variables. In the dark‐blue circle we show an example of how the different variables capture activity levels: mobility (in blue and red) shows greater precision of the movement patterns than movement on the x‐y plane (in yellow).
**Figure S2:** Estimated marginal means (± SE) for low mobility (a) and movement (b) across the exposure treatments (Ctrl, Phe, 2,4‐DCP, Mix).
**Figure S3:** Behavioral variation within individuals (behavioral plasticity; a and c) and between individuals (behavioral individuality; b and d) in low mobility and movement across the exposure treatments (Ctrl: *N* = 58; Phe: *N* = 56; 2,4‐DCP: *N* = 59; Mix: *N* = 59).

## Data Availability

Data and the *R* code used for the statistical analysis of this work are available at https://figshare.com/s/2e9f5251f1a89a7cfdfb.
