# Supplementary material for: Interactive Effects of Two Global Contaminants on Behavioral Variation in Brine Shrimp
Source: Ecol Evol. 2026 Apr 16;16(4):e73495. doi: 10.1002/ece3.73495 (PMC13086631; doi:10.1002/ece3.73495)
Supplement: Supplementary file 1 — Table S1: Presence and concentrations of phenanthrene and 2,4‐dichlorophenol in seawater samples collected from a local natural brine shrimp habitat and adjacent coastal seawaters in Central Italy. Geographic coordinates of sites, dates, and time of collection are reported. Table S2: Concentrations of phenanthrene (Phe) and 2,4‐dichlorophenol (2,4‐DCP) in the water collected from the experimental tanks at the end of the behavioral tests. Table S3: Results from the models for low mobility and movement as the dependent variables. Figure S1: Snapshot of the Integrated Visualization tool in EthoVision XT used to select threshold values for movement and mobility, and to assess the difference between these two variables. In the dark‐blue circle we show an example of how the different variables capture activity levels: mobility (in blue and red) shows greater precision of the movement patterns than movement on the x‐y plane (in yellow). Figure S2: Estimated marginal means (± SE) for low mobility (a) and movement (b) across the exposure treatments (Ctrl, Phe, 2,4‐DCP, Mix). Figure S3: Behavioral variation within individuals (behavioral plasticity; a and c) and between individuals (behavioral individuality; b and d) in low mobility and movement across the exposure treatments (Ctrl: N = 58; Phe: N = 56; 2,4‐DCP: N = 59; Mix: N = 59). [file ECE3-16-e73495-s001.docx]

# SUPPLEMENTARY MATERIAL

# Interactive effects of two global contaminants on behavioral variation in brine shrimp

*Marta Favero^1^, *Bianca Melita Palmas^1^, Giulia Forte^1^, Karina C. Lau^1^, Anisa Bardhi^2^, Andrea Barbarossa^2^, Marialetizia Palomba^1^, Daniele Canestrelli^1^, Giovanni Polverino^1,^*^a^*

^1^Department of Ecological and Biological Sciences, University of Tuscia, Viterbo, Italy

^2^Department of Veterinary Medical Sciences, University of Bologna, Bologna, Italy

*Equally-contributing authors (Marta Favero and Bianca Melita Palmas)

*^a^Corresponding author*

giovanni.polverino@unitus.it; gio.polverino@gmail.com

# Largo dell’Università snc, 01100 Viterbo, Italy

# METHODS

## **Chemical analyses**

We first analyzed concentrations of phenanthrene and 2,4-dichlorophenol in seawater samples collected from a local natural brine shrimp habitat and adjacent coastal seawaters in Central Italy (Table S1). We also analyzed water samples from our own experimental tanks after the behavioral trials ended, to check the concentrations of both contaminants experienced by our animals, following procedures described by Favero and collaborators (2025; Table S2).

For phenanthrene, water samples were first filtered through a fine mesh to remove solid residues and then subjected to liquid-liquid extraction to recover organic compounds with low-medium molecular weight. In particular, 250 mL of sample was extracted three independent times in a liquid-liquid extractor using 250 mL of Ethyl Acetate (EtOAc) or alternatively Dichloromethane (CH₂Cl₂; 250 mL x 3). To reach the maximum extraction efficiency, the pre-treated water was acidified with 0.1N HCl and extracted again with organic solvent (250 mL x 3). The organic phases obtained were then combined and washed with a saturated solution of Sodium Chloride (NaCl), dried over sodium sulphate, filtered, and concentrated under reduced pressure. The obtained samples were then analyzed with gas chromatography and mass spectrometry (GC-MS) using the Agilent COMBO instrument, following derivatization through silylation under standard conditions—i.e., dissolving 2–10 mg of extract in 100 μL of pyridine. We then added 300 μL of bis(trimethylsilyl)trifluoroacetamide with 1% trimethylchlorosilane and 0.390 mg of betulinol to the solution, as an internal standard. The solution was then magnetically stirred at 90°C for four h. The instrument was set with the following analytical conditions: SLB™-5ms capillary column, 30 m x 0.250 mm x 0.250 μm film thickness (catalog no. 28471-U, Supelco), helium flow rate of 1 mL/min, column temperature 100°C (for 2 min) that increased at 5°C/min up to 250°C (maintained for 5 min), and 280°C as the injector temperature. To identify the organic compounds, we used NIST databases, laboratory internal libraries, and compared the fragmentation spectra with authentic standards from the literature. We performed quantitative determination using the internal standard method (betulinol). Each analysis was performed three times independently.

With respect to 2,4-dichlorophenol, a solid-phase extraction (SPE) was performed using Oasis HLB cartridges (6 cc, 200 mg; Waters, Milford, MA, USA). Cartridges were conditioned with 4 mL of methanol followed by 4 mL of ultrapure water (freshly produced in-house using a Sartorius Arium® Ultrapure Water System, Varedo, Italy). A 20 mL aliquot of the water sample was then loaded onto the cartridge. After sample loading, the cartridge was washed with 4 mL of ultrapure water to remove potential interferences. Analytes were eluted with 3 mL of methanol. The eluate was evaporated to dryness under a gentle stream of nitrogen. The dry residue was reconstituted in 200 μL of mobile phase consisting of 10:90 (*v/v*) water:acetonitrile, with acetonitrile (LC–MS/MS grade) purchased from Merck, Milan, Italy. The final extract (7.500 µL) was injected into the LC–MS/MS system for analysis. The analytical system consisted of an Acquity UPLC binary pump (Waters, Milford, MA, USA) coupled to a Xevo TQ-S Micro triple quadrupole mass spectrometer (Waters, Milford, MA, USA). Chromatographic separation was performed using a Waters Acquity UPLC BEH C18 column (50 × 2.100 mm, 1.700 μm), maintained at 40 °C. The mobile phase consisted of water (A) and acetonitrile (B), delivered at a flow rate of 0.300 mL/min under isocratic conditions (10:90, *v/v*) over a 3 min chromatographic run. Mass spectrometric detection was performed in negative electrospray ionization mode (ESI⁻), with a capillary voltage of −1.0 kV. The source temperature was set at 150°C, and the desolvation temperature at 500°C. Cone and desolvation gas flows were maintained at 50 L/h and 900 L/h, respectively, with argon employed as the collision gas. Quantification of 2,4-dichlorophenol was achieved using selected reaction monitoring. The monitored transitions, along with the corresponding cone voltage (CV) and collision energy (CE), were as follows: 161.000 > 124.940 *m/z* (CV: 10 V; CE: 14 eV) and 161.000 > 88.930 *m/z* (CV: 10 V; CE: 20 eV). Data acquisition and processing were carried out using MassLynx 4.2 software (Waters, Milford, MA, USA). To evaluate method performances in terms of specificity, linearity, precision, and accuracy on the day of analysis, water-based calibrators (5 levels ranging from 10 to 200 ng/L) and quality control samples (at 10, 25 and 100 ng/L, each in triplicates) were prepared. The lower limit of quantification, defined as the lowest measured concentration that could be detected with a signal-to-noise (S/N) ratio ≥ 10 and acceptable accuracy and precision (*<*20 %) after injection of four replicates, was 10 ng/L. Method recovery was 92%.

**Table S1** Presence and concentrations of phenanthrene and 2,4-dichlorophenol in seawater samples collected from a local natural brine shrimp habitat and adjacent coastal seawaters in Central Italy. Geographic coordinates of sites, dates, and time of collection are reported.

| **Site** | **Geographic coordinates** | **Date** | **Time** | **Phenanthrene concentration** | **2,4-dichlorophenol concentration** |
| --- | --- | --- | --- | --- | --- |
| Coastal seawater (Civitavecchia’s harbor) | 42°05'17.9'' N, 11°47'31.9'' E | 13/02/23 | 12.45 | 0 µg/L | 0 µg/L |
| Coastal seawater (Civitavecchia’s central) | 42°07'41.8'' N, 11°45'15.4'' E | 31/01/23 | 12.30 | 16.660 µg/L | 69.440 µg/L |
| Natural brine shrimp habitat (Tarquinia saltpans) | 42°11'56.3'' N, 11°42'58.4" E | 07/02/23 | 14.00 | 0 µg/L | 0 µg/L |

**Table S2** Concentrations of phenanthrene (Phe) and 2,4-dichlorophenol (2,4-DCP) in the water collected from the experimental tanks at the end of the behavioral tests.

| **Treatment** | **Initial concentration** | **Concentration after behavioural trials** |
| --- | --- | --- |
| Control | 0 ng/L | 0 ng/L |
| Phe | 400 ng/L | 128 ng/L |
| 2,4-DCP | 400 ng/L | 89 ng/L |
| Mix | 400 ng/L (Phe)  400 ng/L (2,4-DCP) | 128 ng/L (Phe)  68 ng/L (2,4-DCP) |

## **Ethovision XT**

Threshold values for movement (i.e., time spent moving) and mobility were determined using the Integrated Visualization tool in EthoVision XT (see Figure S1). Moreover, this step allowed us to assess different activity levels in *A. parthenogenetica,* differentiating between two dependent variables (movement and mobility)*.* Movement indicated the time spent that an animal spent swimming in the x-y plane. Instead, mobility also included other movements patterns (i.e., rotation, vibration, rolling, or bending of the entire body or specific body parts), which are not detected from the horizontal movements but are common in planktonic organisms (see Figure S1).


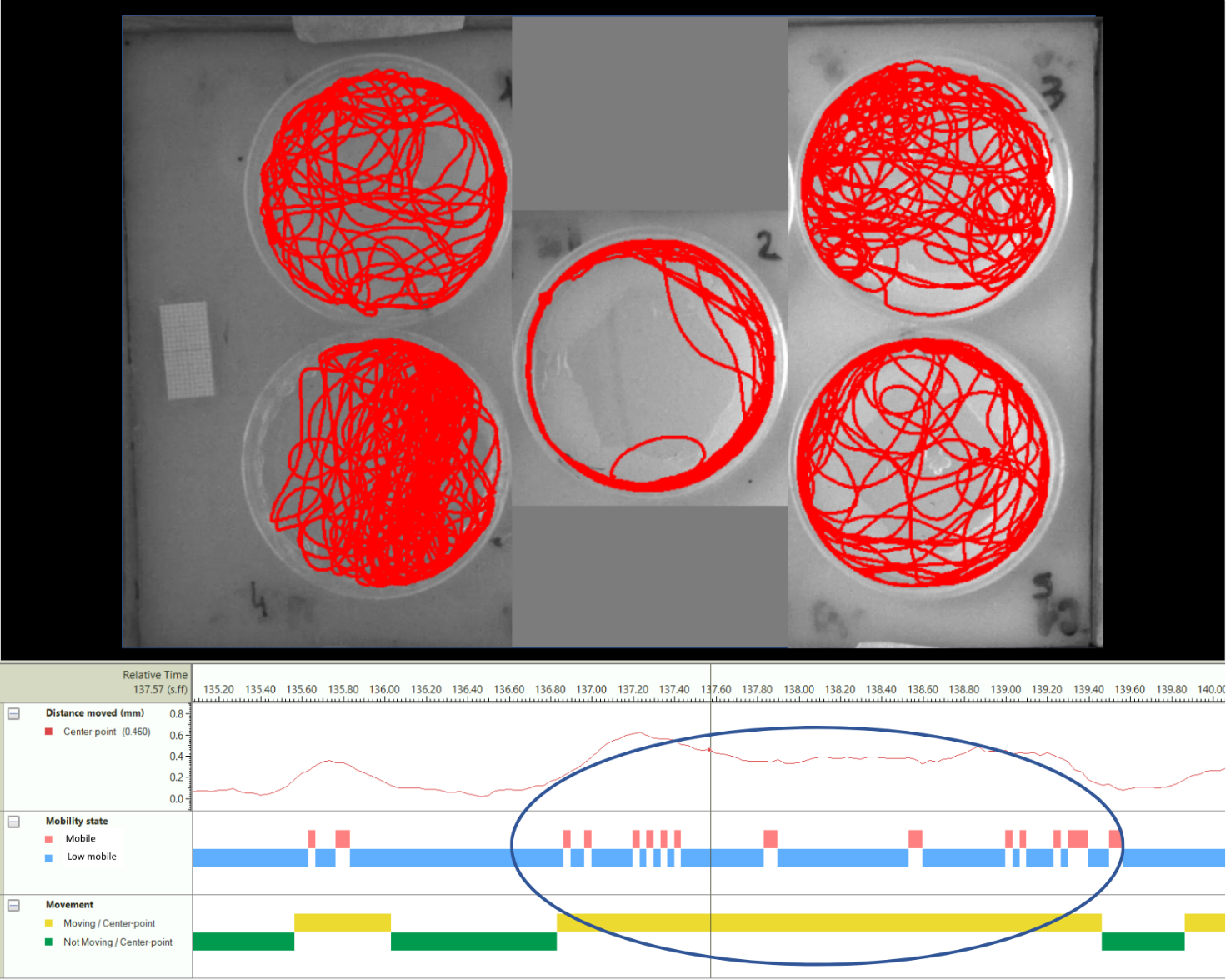


**Figure S1** Snapshot of the Integrated Visualization tool in EthoVision XT used to select threshold values for movement and mobility, and to assess the difference between these two variables. In the dark-blue circle we show an example of how the different variables capture activity levels: mobility (in blue and red) shows greater precision of the movement patterns than movement on the x-y plane (in yellow).

# RESULTS

As observed for mobility levels, results for low mobility revealed significant differences between treatments (*P* = 0.014; Table S3): low mobility increased in individuals exposed to phenanthrene compared to control (Control-Phe: estimate ± SE = −0.549 ± 0.197; *df*_238_; *P* = 0.035; Figure S2a) and Mix treatment (Mix-Phe: estimate ± SE = −0.546 ± 0.196; *df*_242_; *P* = 0.034; Figure S2a). Conversely, individuals exposed to 2,4-dichlorophenol did not differ in their low mobility levels compared to the control group (Control-2,4-DCP: estimate ± SE = −0.226 ± 0.199; *df*_239_; *P* > 0.05; Figure S2a). Moreover, low mobility significantly increased across trials (*P* < 0.001; Table S3). By contrast, average movement (i.e., time spent moving in sec) showed no significant differences between treatment groups (*P* > 0.05; Figure S2b).

At the individual level, our results confirmed a reduction in within-individual variation for low mobility levels in all treatment groups compared to unexposed individuals (Figure S3a). Similarly, we did not find significant differences in between-individual variation regardless of exposure to single pollutants or their combination (Figure S3b). For movement, we observed no significant differences both in variation within- and between-individuals in response to all the exposure treatments (Figure S3c, d).

**Table S3. Results from the models for low mobility and movement as the dependent variables.**

Fixed effects in the models included treatment (Ctrl, Phe, 2,4-DCP, Mix), body size, trial (three repeated measures per individual), experimental arenas (n = 5), and time in zone (recording time of max 600 s). Random effects included random intercepts (individuals and rounds) and slopes (rounds and trials). All models included random intercepts, which allowed variance partitioning: between-individual variation (intercepts; V_between_), within-individual variation (residuals; V_within_), and their proportion (behavioral repeatability). Test statistics and significance levels for random effects were determined by comparing full models to null models (excluding intercepts or slopes) using likelihood ratio tests and Akaike information criteria. The significance level was set at α < 0.05, with significant results highlighted in bold.

| **Low mobility (s)** | | | | | | |
| --- | --- | --- | --- | --- | --- | --- |
| *Fixed effects* | *Sum Sq* | *Mean Sq* | *NumDF* | *DenDF* | *F value* | *P* |
| Treatment | 14.401 | 4.800 | 3 | 247.950 | 3.589 | **0.014** |
| Body size | 1.986 | 1.986 | 1 | 260.010 | 1.485 | 0.224 |
| Trial | 74.524 | 74.524 | 1 | 259.480 | 55.713 | **< 0.001** |
| Arena | 63.503 | 15.876 | 4 | 503.600 | 11.869 | **< 0.001** |
| Time in Zone | 118.440 | 118.440 | 1 | 413.930 | 88.545 | **< 0.001** |
| *Random effects* | *Estimate* | *AIC* | *BIC* | *logLik* | *Chisq* | *P* |
| V_between_ | 3.096 |  |  |  |  |  |
| V_within_ | 1.338 |  |  |  |  |  |
| repeatability | 0.698 | 2246.300 | 2308.500 | −1109.200 | 40.857 | **< 0.001** |
| Round.slope |  | 2235.200 | 2306.300 | −1101.600 | 15.159 | **< 0.001** |
| Trial.slope | 0.188 | 2229.900 | 2301.000 | −1098.900 | 20.454 | **< 0.001** |
| Both slopes |  | 2236.900 | 2316.900 | −1100.500 | 17.394 | **0.002** |
| **Movement (s)** | | | | | | |
| *Fixed effects* | *Sum Sq* | *Mean Sq* | *NumDF* | *DenDF* | *F value* | *P* |
| Treatment | 76.294 | 25.431 | 3 | 367.060 | 1.152 | 0.328 |
| Body size | 4.942 | 4.942 | 1 | 305.920 | 0.224 | 0.636 |
| Trial | 15.101 | 15.101 | 1 | 3.000 | 0.684 | 0.469 |
| Arena | 199.784 | 49.946 | 4 | 694.870 | 2.263 | 0.061 |
| Time in Zone | 139.498 | 139.498 | 1 | 675.980 | 6.319 | **0.012** |
| *Random effects* | *Estimate* | *AIC* | *BIC* | *logLik* | *Chisq* | *P* |
| V_between_ | 0.227 |  |  |  |  |  |
| V_within_ | 22.076 |  |  |  |  |  |
| repeatability | 0.010 | 4307.700 | 4371.500 | −2139.900 | 4.338 | 0.114 |
| Round.slope |  | 4311.700 | 4384.600 | −2139.900 | 0.000 | 1.000 |
| Trial.slope |  | 4310.500 | 4383.500 | −2139.300 | 1.166 | 0.558 |
| Both slopes |  | 4226.900 | 4309.000 | −2095.500 | 88.765 | **< 0.001** |

**
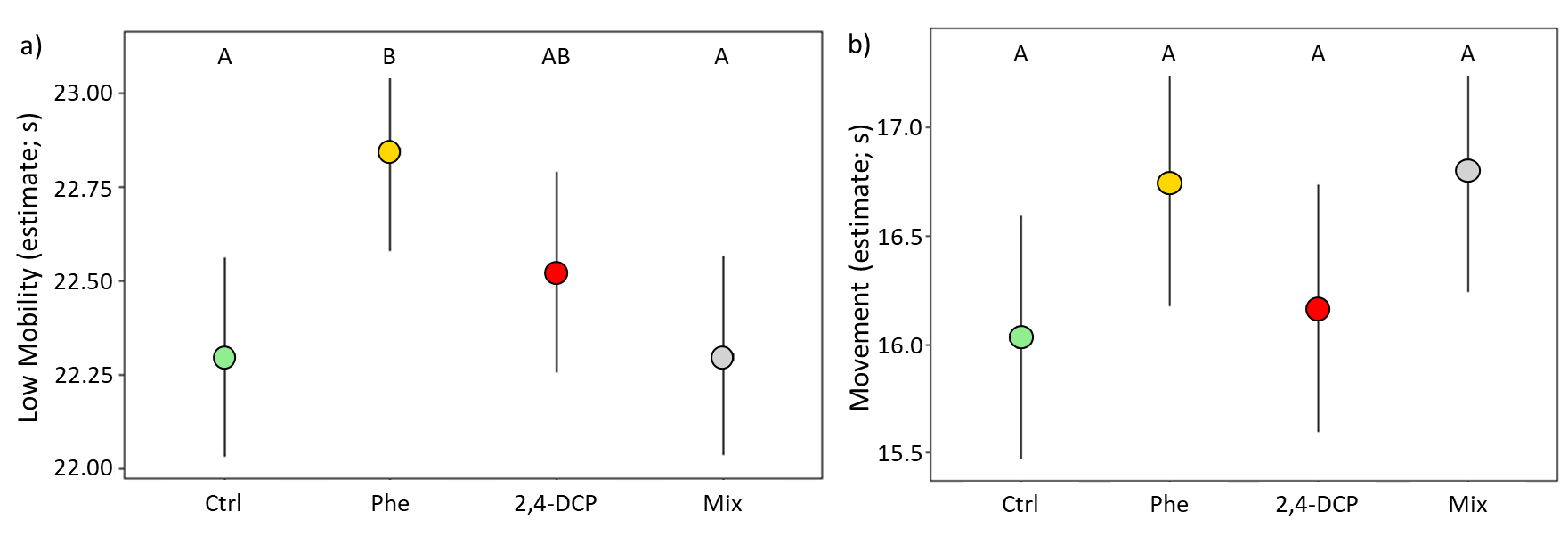
**

**Figure S2. Estimated marginal means (± SE) for low mobility (a) and movement (b) across the exposure treatments (Ctrl, Phe, 2,4-DCP, Mix).**

Pairwise comparisons between treatment groups were adjusted with the Bonferroni method. Treatments not sharing the same letter are significantly different (*P* < 0.05).


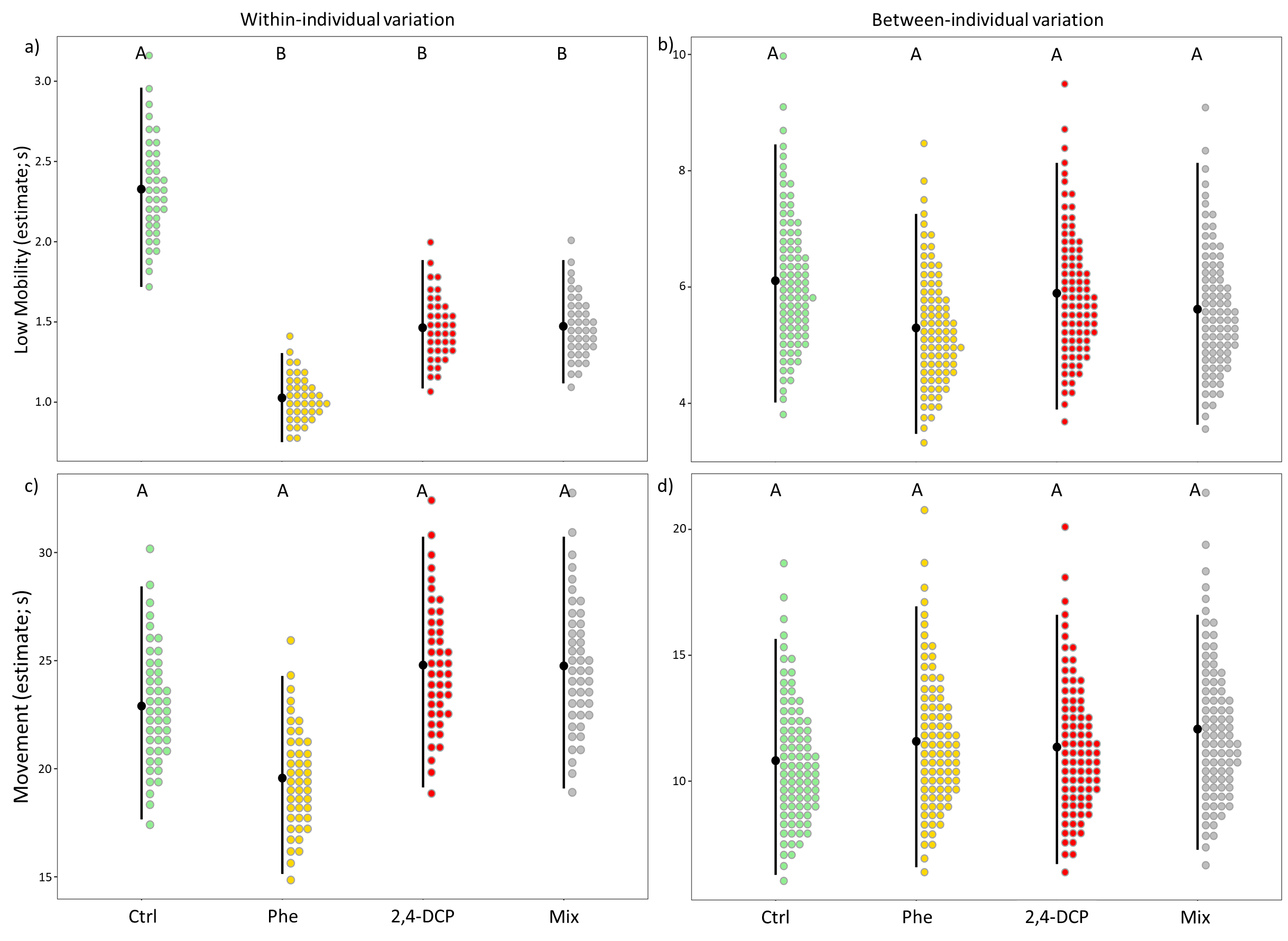


**Figure S3. Behavioral variation within individuals (behavioral plasticity; a, c) and between individuals (behavioral individuality; b, d) in low mobility and movement across the exposure treatments (Ctrl: N = 58; Phe: N = 56; 2,4-DCP: N = 59; Mix: N = 59).**

For each plot, black points represent mean variance estimates, vertical lines represent the 95% credible intervals, and colored dots represent the probability densities.
